# Post-Graduate Medical Education

**Published:** 1921-06-25

**Authors:** 


### POST-GRADUATE MEDICAL EDUCATION.

A report has now been issued by the Committee, under the chairmanship of the Earl of Athlone, which was appointed by Dr. Addison to investigate the needs of medical practitioners and other graduates for further education in medicine in London. On the whole their proposed plan will meet with approval, even though there be some who are pessimistic as to its ultimate materialisation. It is well that the report opens by summarising the many attempts upon and the peculiar difficulties which have in the past surrounded this problem. Clearly the present organisation is far from satisfactory, and much remains to be done before anything like suitable provision can be said to exist. Nevertheless, an opportunity now occurs for this country at last to supply a centre, not only for our own practitioners, but for the Colonies, America, and, to some extent, the mainland of Europe.

Certainly the Metropolis, with its 38,000 beds, possesses a unique supply of material. At the same time, the Committee urges that more use should be made of the cottage hospitals as holding out great possibilities for the instruction of local probationers. It would undoubtedly be advantageous that all doctors within a given area should have facilities, if they wished, to bring their own cases into the wards. In this way better treatment might be obtained, and the practitioner would certainly have opportunity of consulting his professional colleagues.

The chief recommendation, however, and one with which we heartily agree, is for the establishment of a central post-graduate hospital and school, devoted entirely to this work. A new building is not considered necessary. The hospital should be one equipped in up-to-date fashion, and associated with it should be the great special hospitals of the Metropolis. There should certainly be a hospital in London readily adaptable in this way, but there must be a central office also from which the system as a whole can be organised, and where social facilities can be provided.

The Committee's recommendations are thus far in complete accordance with professional views frequently expressed of late, but they go further. Public-health post-graduate work might be concentrated in an Institute of Medicine, directly connected with the University of London and under its administration, occupying a position analogous to that held by the already established Institute of Historical Research.

To those who fear that these things might involve a new and prohibitive expenditure, it is pointed out that the proposed scheme would involve an annual charge of £28,000. Support from local rates would not be expected, as the school and institute, becoming constituent parts of the University of London, would be eligible for inclusion in the list of grant-aided university institutions, for which money is already provided by the Treasury through the University Grants Committee.

